# 769. HIV Pre- and Post-Exposure Prophylaxis (PrEP/PEP) knowledge increases between Academic Curricula in Dominican Republic' Medical Students: A cross-sectional survey.

**DOI:** 10.1093/ofid/ofad500.830

**Published:** 2023-11-27

**Authors:** Leandro Tapia, Hector J Lora, Bismarck S Bisono Garcia, Marcos Nunez, Robert Paulino-Ramírez

**Affiliations:** Instituto de Medicina Tropical & Salud Global, Universidad Iberoamericana, Santo Domingo, Dominican Republic, Santo Domingo, Distrito Nacional, Dominican Republic; Instituto de Medicina Tropical & Salud Global, Universidad Iberoamericana, UNIBE, Santo Domingo, Distrito Nacional, Dominican Republic; Mayo Clinic, Rochester, Minnesota; Universidad Iberoamericana UNIBE, Distrito Nacional, Distrito Nacional, Dominican Republic; Universidad Iberoamericana, Santo Domingo, Distrito Nacional, Dominican Republic

## Abstract

**Background:**

HIV knowledge and prevention strategies, like PrEP/PEP is a core element in current medical practice but represents a challenge for medical students’ curricula. In countries like Dominican Republic (DR), the stigma towards HIV infection restricts the delivery of HIV related information to the general population. Our study aims to understand the level of knowledge between different academical years pose regarding HIV/AIDS, PrEP and PEP and how their curricula content is covered across the years.

**Methods:**

We survey medical students in the DR to measure knowledge on HIV, PrEP/PEP. Students who agreed to participate filled in a survey with 3 sections. Each section had 10 true or false questions. Responses were compared mean knowledge for each topic based on a score out of 10. We analyzed mean scores using ANOVA with Post Hoc analysis, assuming significance when p< 0.05.

**Results:**

A total of 503 participants participated in the survey. ANOVA results showed that there was a statistically significant difference in mean HIV knowledge (F (3, 499) = [25,58], p< 0.001) and PEP knowledge (F (3, 499) = [13,77], p< 0.001) between the academical years. Post-hoc analysis revealed that MS4 had a higher mean score for HIV knowledge (M= 9.3) than MS2 (8.7, p< 0.01) and MS1 (m= 8.2, p< 0.01). Similarly, MS3 had a higher mean HIV knowledge than MS2 (p< 0.01) and MS1 (p< 0.01). Likewise, MS2 had an increased mean HIV knowledge when compared to MS1 (p< 0.01). When we compare mean PEP knowledge, we found that MS3 (m= 7.45) had increased knowledge than MS1 (6.4, p< 0.01) and MS2 (m= 6.9, p< 0.05). Similarly, we found that MS4 had increased mean PEP Knowledge (m= 7.5) when compared to MS1 and MS2 (p< 0.001). When we studied mean PrEP knowledge, ANOVA analysis found no significant difference (F (3, 499) = [1,24], p= 0.29).

**Conclusion:**

Our study suggests that the mean knowledge of HIV and PEP increases throughout the academic progression of medical school. Although there is no difference in PEP knowledge, this may suggest that medical schools are teaching PEP during MS3-MS4 cycle rather than the M1-M2 cycle. Also, the homogenous mean PrEP knowledge could suggest that the students are learning from non-academic sources. This emphasizes the curricular designers of medical schools to review the contents on prevention of communicable diseases.

**Disclosures:**

**All Authors**: No reported disclosures

